# Factors influencing the first thousand days of life

**DOI:** 10.1093/eurpub/ckac129.671

**Published:** 2022-10-25

**Authors:** E Capitani, C Lorenzini, A Biuzzi, L Alaimo, G Messina, N Nante

**Affiliations:** 1 Post Graduate School of Public Health, University of Siena, Siena, Italy; 2 Degree Course in Obstetrics, University of Siena, Siena, Italy; 3 Department of Molecular and Developmental Medicine, University of Siena, Siena, Italy

## Abstract

**Background:**

The first 1000 days are crucial for the health of the baby and the well-being of the mother-baby dyad, which forms a single complex organism with its co-metabolism expressed through sophisticated neurobiological, epigenetic and microbiome development mechanisms. This study aims to investigate how much social support can influence the path of pregnancy and motherhood.

**Methods:**

The retrospective observational study was conducted on a sample of mothers enrolled through social networks who were administered a questionnaire from July to September 2021. The questionnaire consisted of 37 questions: 11 analyzed sociodemographic variables, 20 were on current / any previous pregnancies and breastfeeding, and 6 were used to calculate the Maternity Social Support Scale (MSSS-Webster et al.). STATA 14 was used for statistical analysis.

**Results:**

Our sample consisted of 3447 women. 88.0% wanted the pregnancy, and 63.5% planned it. The average of the Maternity Social Support Scale (MSSS) was 23.91 points. A low MSSS score correlates with a higher risk of cessation of breastfeeding before 6 months of age, a higher risk of not having spontaneous labour, a higher risk of cesarean section and a higher risk of not having a spontaneous birth. On the other hand, a higher MSSS total score is a protective factor concerning breastfeeding duration, which is more likely to be longer-lasting (>6 months), to have spontaneous onset labour with a higher probability of spontaneous delivery.

**Conclusions:**

The results showed that most of our sample have good friends who support them, can often count on their family, and receive help from their partner/husband. The outcomes of pregnancy, childbirth and motherhood are strongly influenced and conditioned by the social context in which they occur and the support the woman can receive. The presence or lack of this support can affect the health of newborns.

**Key messages:**

• The first 1,000 days is a vulnerable phase in which parents, institutions and health professionals should create early interventions for the proper development and promotion of good health.

• the outcomes of pregnancy, birth and motherhood are strongly influenced and conditioned by the social context, but especially by the presence or lack of support that can affect the health of newborns.

